# Effects of finerenone and glucagon-like peptide 1 receptor agonists on cardiovascular and renal outcomes in type 2 diabetes mellitus: a systematic review and meta-analysis

**DOI:** 10.1186/s13098-023-01251-2

**Published:** 2024-01-11

**Authors:** Xia Gu, Shimin Jiang, Yue Yang, Wenge Li

**Affiliations:** 1 China-Japan Friendship Hospital (institute of Clinical Medical Sciences), Chinese academy of Medical Sciences & Peking union Medical College, Beijing, China; 2 Department of Nephrology, China-Japan Friendship Hospital, Beijing, China

**Keywords:** Finerenone, Glucagon-like peptide 1 receptor agonists, Meta-analysis, Type 2 Diabetes Mellitus

## Abstract

**Objective:**

To assess the effects of finerenone and glucagon-like peptide 1 receptor agonists (GLP1-RA) on cardiovascular and renal outcomes in patients with type 2 diabetes mellitus (T2DM), and the relative cardiovascular benefits in patients with or without established atherosclerotic cardiovascular disease for different outcomes with these classes of drugs.

**Methods:**

We searched PubMed, the Cochrane Library, and Embase from January 1, 2000, to December 30, 2022, to identify randomized controlled trials. The primary outcomes were the composite of nonfatal myocardial infarction, nonfatal stroke, and cardiovascular death (MACE); hospitalization for heart failure (HHF); and a composite of renal outcomes. The results were reported as hazard ratios (HRs) with 95% confidence intervals (CIs).

**Results:**

In total, we identified 11 trials and 73,927 participants, 13,847 (18.7%) in finerenone trials and 60,080 (81.3%) in GLP1-RA trials. Finerenone reduced the risk of MACE by 13% (HR, 0.87; 95% CI, 0.79–0.95; *P* = 0.003), while GLP1-RA reduced the risk in a similar magnitude by 13% (HR, 0.87; 95% CI, 0.83–0.92; *P* < 0.001). For both drug classes, the effect on lowering the risk of MACE was restricted to approximately 14% in patients with established atherosclerotic cardiovascular disease (HR, 0.86; 95% CI, 0.82–0.90; *P* < 0.001), whereas no effect was observed in patients without established atherosclerotic cardiovascular disease (HR, 0.93; 95% CI, 0.85–1.02; *P* = 0.12). GLP1-RA reduced myocardial infarction, stroke and cardiovascular death more than finerenone (which appeared to have no effect). Only finerenone was beneficial for reducing the risk of HHF (HR, 0.78; 95% CI, 0.66–0.92; *P* = 0.003). Both finerenone (HR, 0.84; 95% CI, 0.77–0.92; *P* < 0.001) and GLP1-RA (HR, 0.81; 95% CI, 0.76–0.86; *P* < 0.001) reduced the risk of kidney disease progression, including macroalbuminuria, and finerenone was superior to GLP1-RA in delaying deterioration of kidney function.

**Conclusions:**

Finerenone and GLP1-RA lead to a risk reduction in MACE to a similar degree in patients with established atherosclerotic cardiovascular disease. For both drug classes, the effect on lowering the risk of progression of kidney disease was also in a similar magnitude in patients with T2DM, whereas only finerenone had a significant protective effect against HHF. Treatment decisions for patients with T2DM should consider the clinical benefit profiles of each drug.

**Supplementary Information:**

The online version contains supplementary material available at 10.1186/s13098-023-01251-2.

## Introduction

Over the past few decades, the prevalence of type 2 diabetes mellitus (T2DM) has risen dramatically worldwide, and approximately 537 million adults are living with diabetes [1], resulting in an elevated risk of cardiovascular and renal diseases. As the prevalence of T2DM increases, it has become one of the leading causes of the substantial increase in end-stage renal disease (ESRD) globally. Even with the current treatment options available, people with T2DM still face a considerable risk of developing cardiovascular and renal events such as myocardial infarction, stroke and ESRD [2]. Therefore, the prevention of cardiovascular diseases (CVD) and DKD progression is critical for the management of patients with T2DM.

Recently, trials on two new classes of antidiabetic agents, finerenone and glucagon-like peptide 1 receptor agonists (GLP1-RA), have shown cardiovascular and kidney benefits in patients with T2DM. Notably, in clinical trials of renin-angiotensin system (RAS) inhibitors therapy in people with type 2 diabetes, lowering albuminuria to levels < 300 mg/g creatinine or by > 30% from baseline has been related to improved renal and cardiovascular outcomes, leading to the ADA’s clinical practice recommendation that RAS inhibitors should be the first-line drug therapy in diabetes [3]. Therefore, it should be mentioned that the benefit of these two new classes of glucose-lowering medications was on background RAS inhibitors therapy in > 80% of the participants. Finerenone is a novel selective and nonsteroidal mineralocorticoid receptor antagonist. Compared with steroidal mineralocorticoid receptor antagonists, finerenone has shown more potent anti-inflammatory and anti-fibrotic effects in rodent models [4–6]. Two large-scale randomized placebo-controlled trials targeted at patients with T2DM and chronic kidney disease (CKD) have shown that finerenone significantly lowers the occurrences of composite cardiovascular outcome (defined as a composite of nonfatal myocardial infarction, nonfatal stroke, and cardiovascular death) and composite renal outcome (defined as a composite of a sustained decrease of at least 40% in the estimated glomerular filtration rate (eGFR) from the baseline, kidney failure, or death from renal causes), irrespective of history of CVD [7, 8].

There is good evidence to support the use of GLP1-RA. Several sizable randomized placebo-controlled trials (RCTs) have shown cardiovascular and renal benefits in patients with T2DM and/or CKD. In addition, the ADA recommended a GLP1-RA or sodium-glucose co-transporter-2 (SGLT2) inhibitors for individuals with T2DM who have established atherosclerotic cardiovascular disease or indicators of high cardiovascular risk, CKD, or heart failure to reduce the risk of MACE [9].

A recently published meta-analysis reported a significant reduction in MACE with the use of GLP1-RA in patients with T2DM [10, 11]. However, to date, the impact of finerenone and GLP1-RA on MACE in patients with or without established atherosclerotic cardiovascular disease (ASCVD) has not been confirmed. As such, we designed the present meta-analysis based on RCTs to compare the clinical benefit of finerenone and GLP1-RA in patients with T2DM with and without established ASCVD and to update their overall cardiovascular and renal effects.

## Methods

### Registration

This systematic review was registered with PROSPERO (CRD42023405275).

### Literature search and study selection

The search strategy was performed in accordance with the Preferred Reporting Items for Systematic Review and Meta-Analysis Protocols (PRISMA-P) [12–14]. A systematic search of randomized, placebo-controlled, cardiovascular or kidney outcome trials of finerenone and GLP1-RA was performed using PubMed, the Cochrane Library, and Embase from January 1, 2000, to December 30, 2022. For English-language publications, search terms including “finerenone”, “glucagon-like peptide-1 receptor agonist”, “type 2 diabetes mellitus” and related phrases, the names of drugs, and terms related to randomized, controlled trials were used. The details of the search algorithm are presented in the Additional file 1. Studies including individuals with type 1 diabetes mellitus (T1DM) and participants ≤ 18 years of age were excluded. A literature search and screening were performed to identify relevant studies by two independent authors (G and J), and any discrepancies in the eligibility of studies were resolved by consulting with a third author (L).

### Outcomes

Patients were stratified into those with established ASCVD (Table S1 shows this in more detail [see Additional file 1]). Efficacy outcomes of interest included MACE, HHF, and renal outcomes. MACE was defined as a composite of non-fatal myocardial infarction, non-fatal stroke, and cardiovascular death (as for finerenone trials, MACE was defined as a composite of non-fatal myocardial infarction, non-fatal stroke, cardiovascular death and hospitalization for heart failure). Total myocardial infarction and stroke were used instead when data on non-fatal myocardial infarction and non-fatal stroke were not available. The definition of renal outcomes was a broad composite of new-onset persistent macroalbuminuria, persistent doubling of the serum creatinine level or a decline in eGFR of at least 40% from baseline, the need for kidney replacement therapy, or death from renal causes (Table S2 shows this in more detail [see Additional file 1]) and worsening of kidney function, including either sustained doubling of serum creatinine or at least a 40% decline in eGFR from baseline.

### Data extraction and quality assessment

The data extraction was performed by two independent authors (G and J). The following data were extracted: study characteristics (trial, age, sex, BMI, HbA1c, eGFR, duration of diabetes and median follow-up year), description of intervention (drug class, name, dose), and outcomes. Risk of bias assessment was conducted by two independent authors (G and Y) using the Cochrane tool for assessing risk of bias in randomized clinical trials (RoB 2.0) [15]. Any discrepancies in data extraction and risk-of-bias assessment were resolved by consulting a third author (L). We did not assess publication bias due to the limited number of eligible studies.

### Statistical analysis

Summary statistics from the individual trials included were used due to the lack of individual-level data. Hazard ratios (HRs) with 95% confidence intervals (CIs) from the trial papers, supplementary appendices, or secondary publications were used to estimate the efficacy of treatments for dichotomous outcomes. For all outcomes, heterogeneity between studies was assessed using Cochrane’s Q test and Higgins and Thompson’s I^2^. Cochran’s Q statistic *p* values less than 0.05 were considered significant for heterogeneity. Heterogeneity was considered to indicate a low, moderate, or high likelihood of differences between studies if the I^2^ value was 25%, 50%, or 75%, respectively [16]. For each study, estimates were combined by use of inverse variance-weighted averages of logarithmic HRs in fixed-effects models. Random-effects models with the use of restricted maximum likelihood and Hartung-Knapp adjustment were considered to estimate the treatment effect by drug class [17]. All analyses were performed with R (version 4.3.0, R Core Team, 2023) and the R package “metafor” (version 4.2-0) [18].

## Results

### Baseline characteristics of the included trials

**Fig. 1:**
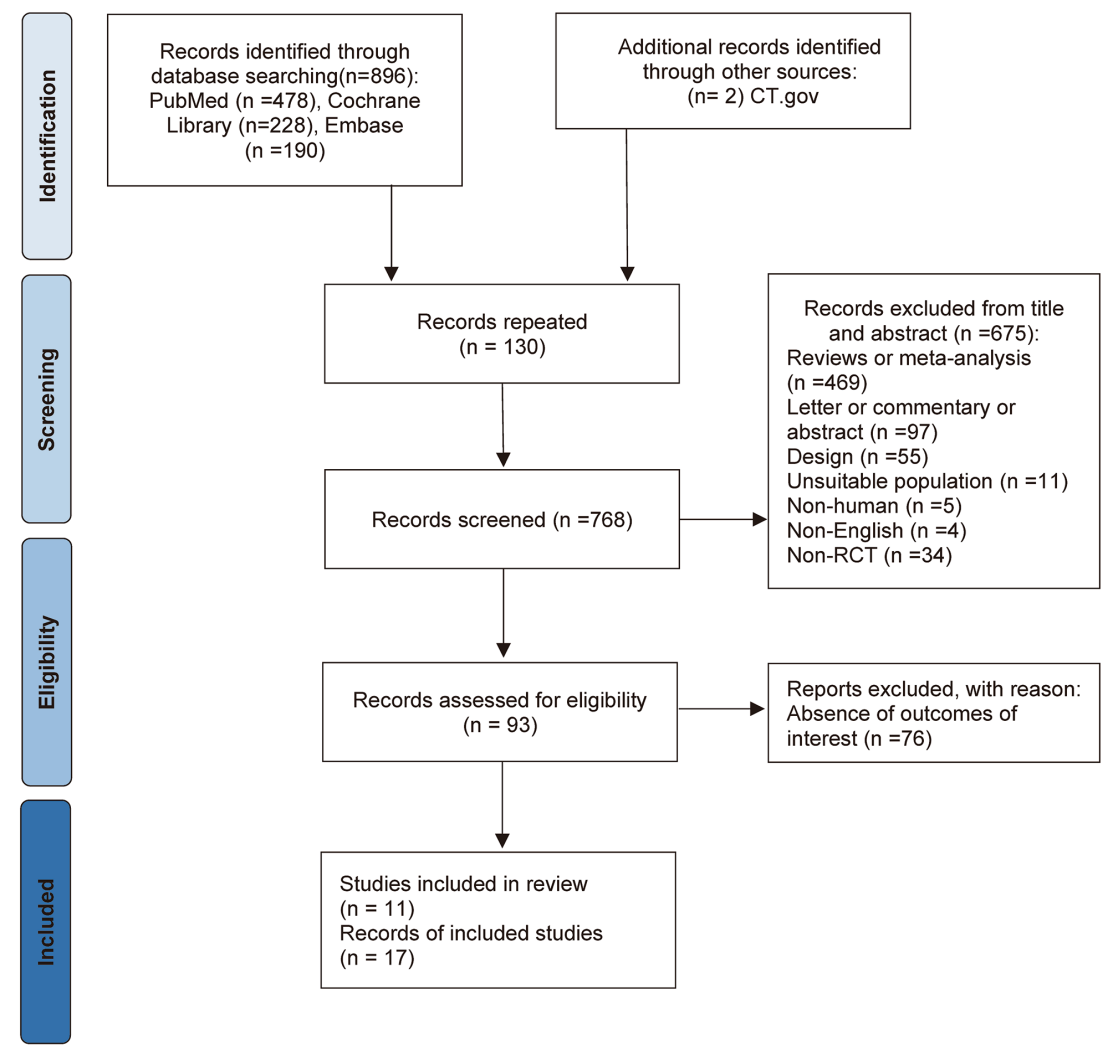
PRISMA flowchart: study selection

We identified 896 records through a database search of PubMed (n = 478), Cochrane Library (n = 228), and Embase (n = 190) in the primary search. An overview of the search and the selection process is shown in Fig. 1. Of 17 full-text articles assessed after screening, we identified a total of 11 large-scale randomized controlled trials, 3 finerenone trials [7, 8, 19], and 8 GLP1-RA trials [20–29] that were eligible for inclusion (Table 1). In total, we identified 73,927 randomly assigned participants, 13,847 (18.7%) participants in finerenone trials and 60,080 (81.3%) participants in GLP1-RA trials. The mean age of patients ranged from 59 to 66 years across the trials, and 47,877 (64.8%) participants were women. The proportion of participants with an eGFR less than 60 mL/min per 1.73 m² ranged from 21.6 to 40.0% across the trials, with the exception of FIDELIO-DKD (Finerenone in Reducing Kidney Failure and Disease Progression in Diabetic Kidney Disease), which had a substantially larger proportion (88.4%).

**Table 1:** Baseline characteristics of included trials

Trial	Drug	Drug dose(mg/d)	Trial participants, n			Age (years)		Gender (male), n (%)		BMI	
			Total	I	C	I	C	I	C	I	C
***Finerenone vs. placebo***											
FIDELIO-DKD	Finerenone	10/20	5674	2833	2841	65.4 ± 8.9	65.7 ± 9.2	1953 (68.9)	2030 (71.5)	31.1 ± 6.0	31.1 ± 6.0
FIGARO-DKD	Finerenone	10/20	7352	3686	3666	64.1 ± 9.7	64.1 ± 10.0	2528 (68.6)	2577 (70.3)	31.5 ± 6.0	31.4 ± 5.9
ARTS-DN	Finerenone	1.25/2.5/5/7.5/10/15/20	823	729	94	64.33 ± 9.20	63.26 ± 8.68	570 (78.4)	69 (73.4)	31.75 ± 5.57	32.49 ± 5.27
***GLP-1RA vs. placebo***											
ELIXA	Lixisenatide	0.01	6068	3034	3034	59.9 ± 9.7	60.6 ± 9.6	2111 (69.6)	2096 (69.1)	30.1 ± 5.6	30.2 ± 5.8
LEADER	Liraglutide	1.8	9340	4668	4672	64.2 ± 7.2	64.4 ± 7.2	3011 (64.5)	2992 (64.0)	32.5 ± 6.3	32.5 ± 6.3
SUSTAIN-6	Semaglutide	0.5/1 weekly	3297	1648	1649	64.7 ± 7.2	64.6 ± 7.5	1013 (61.5)	989 (60.0)	32.8 ± 6.2	32.8 ± 6.2
EXSCEL	Exenatide	2 weekly	14,752	7356	7396	61.8 ± 9.4	61.9 ± 9.4	4562 (62.0)	4587 (62.0)	32.7 ± 6.4	
HARMONY	Albiglutide	30/50	9463	4731	4732	64.1 ± 8.7	64.2 ± 8.7	3304 (70.0)	3265 (69.0)	32.3 ± 5.9	32.3 ± 5.9
REWIND	Dulaglutide	1.5 weekly	9901	4949	4952	66.2 ± 6.5	66.2 ± 6.5	2643 (53.4)	2669 (53.9)	32.3 ± 5.7	32.3 ± 5.8
AMPLITUDE-O	Exenatide	2 weekly	4076	2717	1359	64.6 ± 8.2	64.4 ± 8.3	1792 (66.0)	940 (69.2)	32.9 ± 6.2	32.4 ± 6.0
PIONEER 6	Semaglutide	14	3183	1591	1592	66 ± 7	66 ± 7	1084 (68.1)	1092 (68.6)	32.3 ± 6.6	32.3 ± 6.4

I: intervention, C: control, N/A: not available

**Table 1:** (Continued)

Trial	HbA1C (%)		eGFR (ml/ min/1.73 m^2^)		Duration of diabetes (years)		Median follow year (years)	eGFR < 60 ml/min per 1.73 m^2^, n (%)	Proportion of patients with established atherosclerotic cardiovascular disease, n (%)
	I	C	I	C	I	C			
***Finerenone vs. placebo***									
FIDELIO-DKD	7.7 ± 1.3	7.7 ± 1.4	44.4 ± 12.5	44.3 ± 12.6	16.6 ± 8.8	16.6 ± 8.8	2.6	5016 (88.4)	2605 (45.9)
FIGARO-DKD	7.7 ± 1.4	7.7 ± 1.4	67.6 ± 21.7	68.0 ± 21.7	14.5 ± 8.6	14.4 ± 8.4	3.4	2812 (38.3)	3330 (45.3)
ARTS-DN	7.6 ± 1.3	7.6 ± 1.3	66.9 ± 21.9	72.2 ± 20.4	N/A		N/A	328 (40.0)	N/A
***GLP-1RA vs. placebo***									
ELIXA	7.7 ± 1.3	7.6 ± 1.3	76.7 ± 21.3	75.2 ± 21.4	9.2 ± 8.2	9.4 ± 8.3	2.1	1407 (23.2)	6068 (100.0)
LEADER	8.7 ± 1.6	8.7 ± 1.5	80.2 ± 27.2	80.5 ± 10.8	12.8 ± 8.0	12.9 ± 8.1	3.8	2158 (23.1)	7598 (81.3)
SUSTAIN-6	8.7 ± 1.5	8.7 ± 1.5	75.9 ± 25.9	76.4 ± 27.2	14.2 ± 8.2	13.6 ± 8.0	2.1	939 (28.5)	2735 (83.0)
EXSCEL	8.1 ± 1.0		78.4 ± 24.1		13.1 ± 8.3		3.2	3191 (21.6)	10,782 (73.1)
HARMONY	8.76 ± 1.5	8.72 ± 1.5	79.1 ± 25.6	78.9 ± 25.4	14.1 ± 8.6	14.2 ± 8.9	1.6	N/A	9463 (100.0)
REWIND	7.3 ± 1.1	7.4 ± 1.1	77.2 ± 22.7	76.6 ± 22.8	10.5 ± 7.3	10.6 ± 7.2	5.4	2199 (22.2)	3114 (31.5)
AMPLITUDE-O	8.90 ± 1.46	8.94 ± 1.52	72.2 ± 21.9	72.9 ± 23.3	15.6 ± 8.8	15.1 ± 8.7	1.81	N/A	3650 (89.6)
PIONEER 6	8.2 ± 1.6	8.2 ± 1.6	74 ± 21	74 ± 21	14.7 ± 8.5	15.1 ± 8.5	N/A	856 (26.9)	2695 (84.7)

I: intervention, C: control, N/A: not available

### Risk of bias

**Fig. 2:**
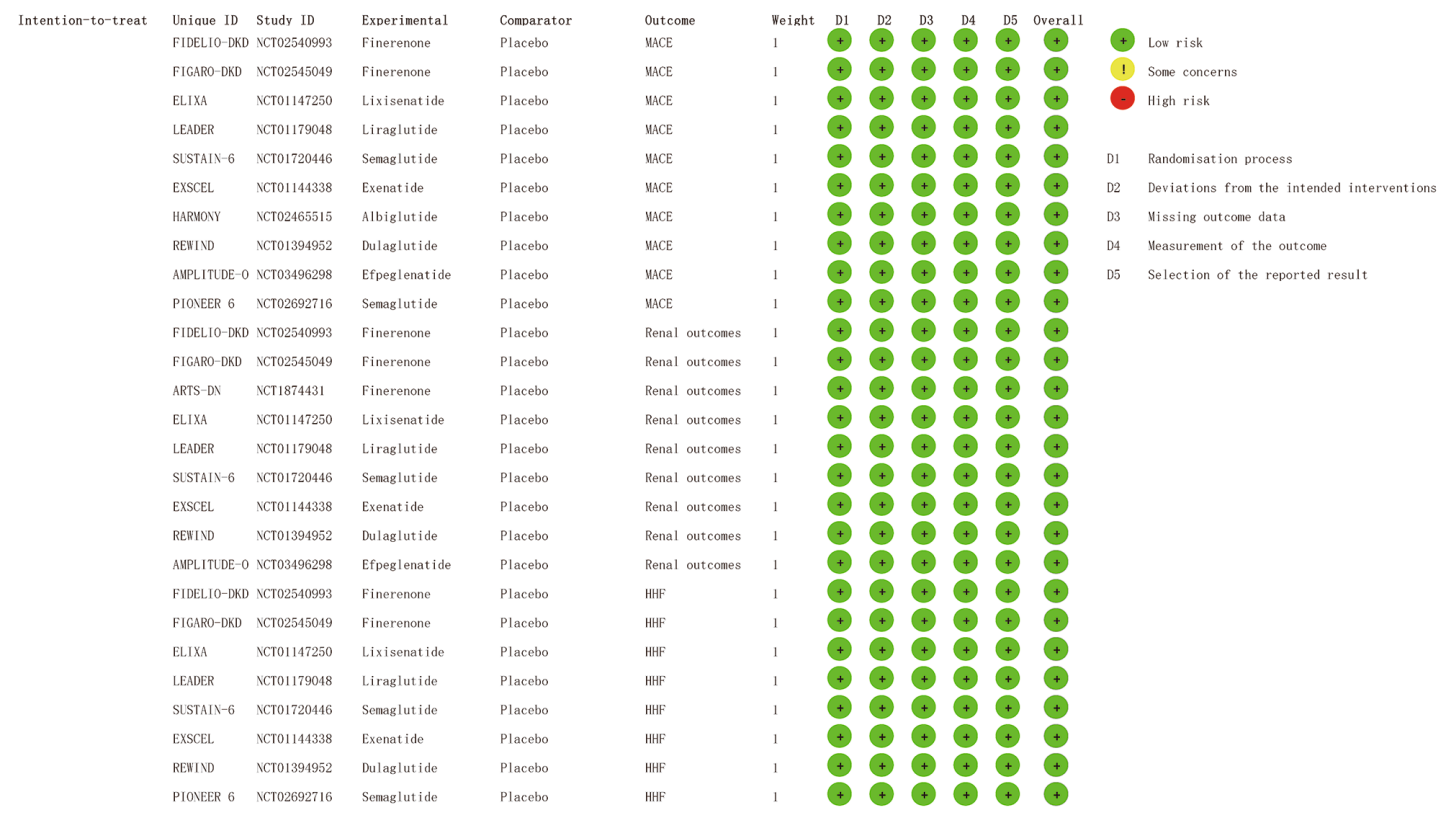
Detailed risk of bias in each trial

We assessed the risk of bias using the Cochrane Collaboration Risk-of-Bias tool (RoB 2.0) for randomized trials. Additional file 2 presents the risk of bias in each trial. The quality evaluation of the included studies is shown in Fig. 2. All included trials were deemed high quality and at low risk of bias in 3 outcomes.

### MACE

Across all included trials, 8343 of 73,106 participants (11.4%) experienced a MACE event (1764 patients in the finerenone trials and 6579 patients in the GLP1-RA trials). A total of 79.0% of the MACE events occurred in the group with established ASCVD.

**Fig. 3:**
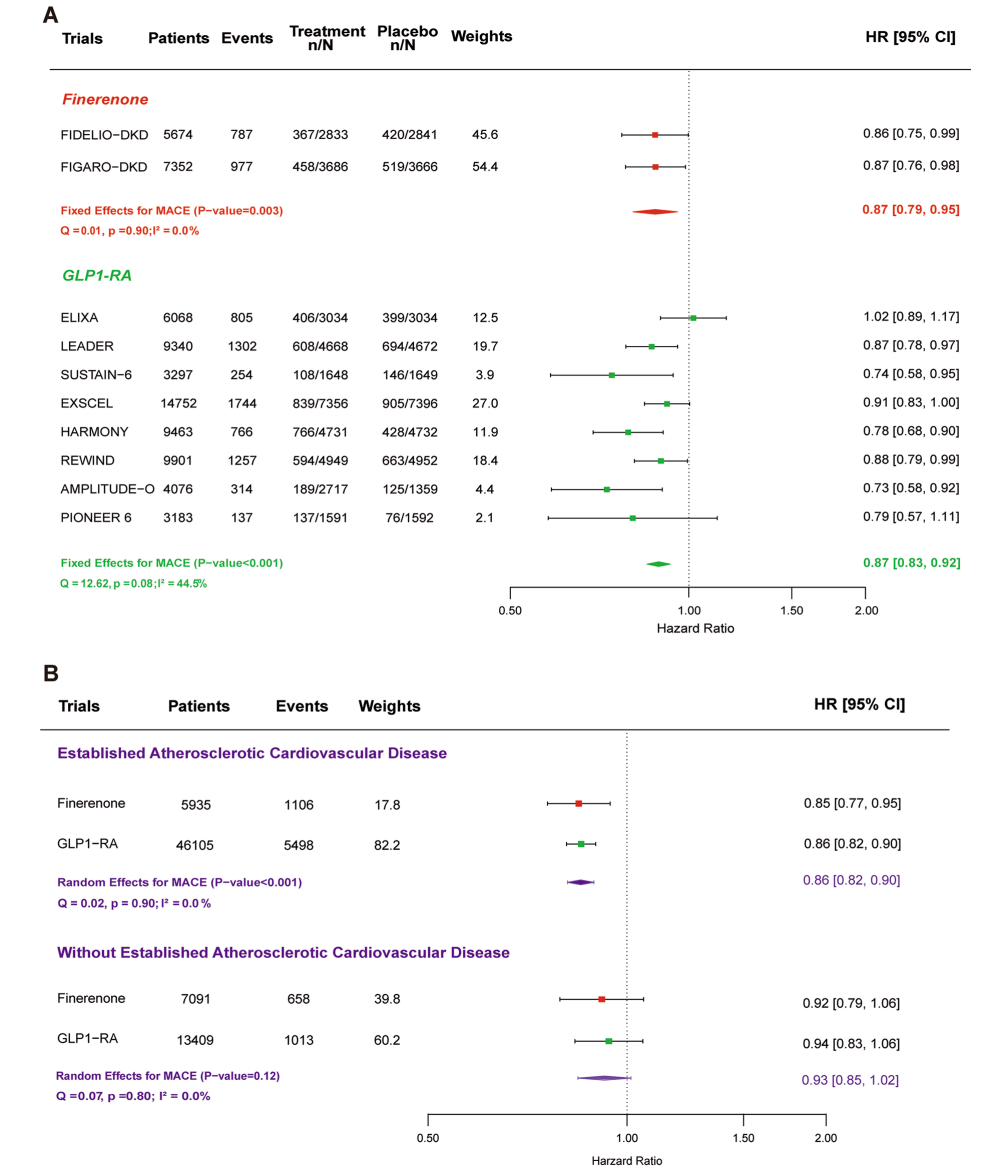
Meta-analysis of finerenone and GLP1-RA trials on MACE. **(A)** Meta-analysis of finerenone and GLP1-RA trials on MACE stratified by drug class. **(B)** Meta-analysis of finerenone and GLP1-RA trials on MACE stratified by established atherosclerotic cardiovascular disease (ASCVD). Forest plot showing the treatment estimates of each drug class in each subgroup using random effects

Overall, both drug classes have significant effects on lowering the risk of MACE in T2DM by a similar magnitude. Compared with placebo, finerenone decreased the risk of MACE by 13% (HR, 0.87; 95% CI, 0.79–0.95; *P* = 0.003), and the effect of finerenone on MACE was consistent across studies (I^2^ = 0.0%, P for heterogeneity, 0.32). Treatment with GLP1-RA did significantly reduce the risk of MACE by 13% (HR, 0.87; 95% CI, 0.83–0.92; *P* < 0.001; P for heterogeneity, 0.08) (Fig. 3A). In FIGARO-DKD trial, the cardiovascular benefits of finerenone therapy were consistent across categories according to the baseline eGFR [8]. GLP1-RA did not reduce the MACE risk in patients with severe kidney disease (baseline eGFR < 60mL/min/1.73m^2^) but reduce the risk of MACE in moderate kidney disease (baseline eGFR ≥ 60mL/min/1.73m^2^) (Figure S1 shows this in more detail [see Additional file 1]). For both drug classes, the effect on lowering the risk of MACE was restricted to approximately 14% in patients with established ASCVD (HR, 0.86; 95% CI, 0.82–0.90; *P* < 0.001), with nearly identical effects for finerenone (HR, 0.85; 95% CI, 0.77–0.95) and GLP1-RA (HR, 0.86; 95% CI, 0.82–0.90), whereas in the trials published to date, neither reduces the risk of MACE in patients without established ASCVD (HR, 0.93; 95% CI, 0.85–1.02; *P* = 0.12). (Fig. 3B; Figure S2 shows this in more detail [see Additional file 1]).

### Treatment effect on the individual components of MACE

A total of 3703 patients experienced a myocardial infarction (3341 patients in GLP1-RA trials and 362 patients in finerenone trials), 2042 experienced a stroke (1646 patients in GLP1-RA trials and 396 patients in finerenone trials), and 3395 experienced a cardiovascular death (2709 in GLP1-RA trials and 686 patients in finerenone trials). Only GLP1-RA reduced the relative risk of myocardial infarction by 9% (HR, 0.91; 95% CI, 0.85–0.98; *P* = 0.008; P for heterogeneity, 0.13), whereas finerenone had no effect on lowering the relative risk of myocardial infarction (HR, 0.90; 95% CI, 0.74–1.11; *P* = 0.34; P for heterogeneity, 0.32; Figure S3 shows this in more detail [see Additional file 1]). Similarly, GLP1-RA is the only drug class that convincingly reduces the relative risk of stroke and cardiovascular death by 17% (HR, 0.83; 95% CI, 0.76–0.92; *P* < 0.001; P for heterogeneity, 0.77) and 13% (HR, 0.87; 95% CI, 0.81–0.94; *P* < 0.001; P for heterogeneity, 0.33), respectively, whereas finerenone has no effect (Figure S4 and S5 shows this in more detail [see Additional file 1]).

### HHF

**Fig. 4:**
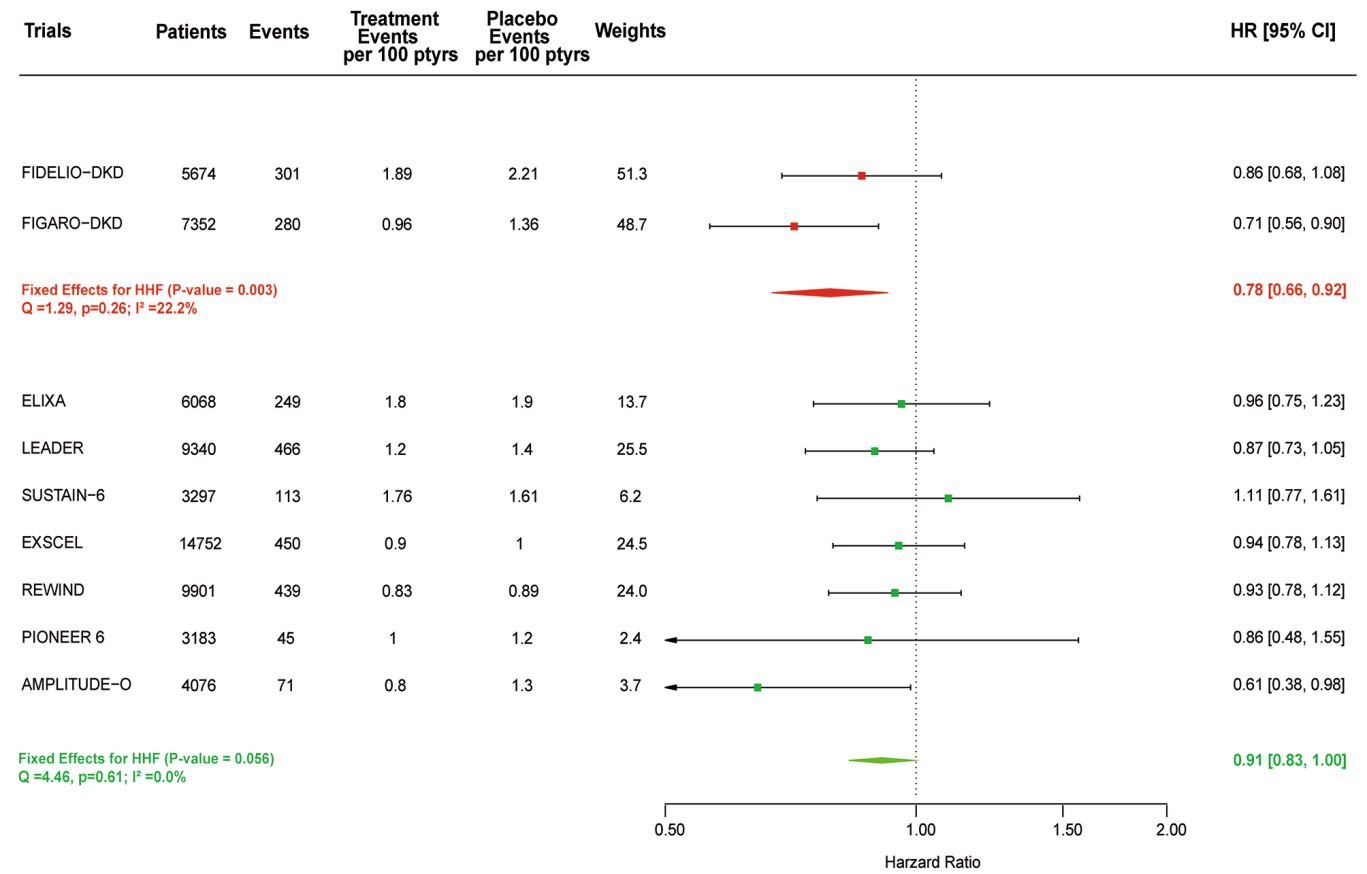
Meta-analysis of finerenone and GLP1-RA trials on HHF stratified by drug class

Overall, HHF occurred in a total of 2414 participants, 581 in the finerenone trials and 1833 in the GLP1-RA trials (not including data from the HARMONY trial that did not directly report that outcome). Treatment with GLP1-RA might have no effect on lowering the relative risk of HHF (HR, 0.91; 95% CI, 0.83–1.00; *P* = 0.056), and the effect of GLP1-RA on HHF was consistent across studies (I^2^ = 0%, P for heterogeneity, 0.53). A sensitivity analysis yielded an almost identical effect estimate (HR, 0.90; 95% CI, 0.83–0.98; *P* = 0.02). Finerenone reduced the relative risk for HHF by 22% (HR, 0.78; 95% CI, 0.66–0.92; *P* = 0.003; P for heterogeneity, 0.26; I^2^ = 22.2%; Fig. 4). The effect of GLP1-RA and SGLT2 inhibitors to reduce HHF and other risks associated with a history of heart failure (HF) has been assessed in newly released publications [30, 31]. Compared with placebo, GLP1-RA did not reduce the risk of HHF or the composite of HHF or cardiovascular death in patients with HF history but reduced these outcomes in patients without HF history. In the present analysis, finerenone reduce HHF in patients with HF history (HR, 0.68; 95% CI, 0.47–0.96; *P* = 0.036; P for heterogeneity, 0.91; I^2^ = 0.0%) and showed a tendency for reduction of HHF in patients without HF history (HR, 0.83; 95% CI, 0.69–1.00; *P* = 0.058; P for heterogeneity, 0.16; I^2^ = 49.9%). Compared with placebo, finerenone did not reduce the composite of HHF or cardiovascular death in patients with HF history (HR, 0.82; 95% CI, 0.62–1.08; *P* = 0.162; *P* for heterogeneity, 0.86; I^2^ = 0.0%) but reduced this outcome in patients without HF history (HR, 0.86; 95% CI, 0.76–0.97; *P* = 0.015; P for heterogeneity, 0.57; I^2^ = 0.0%) (Figure S6 shows this in more detail [see Additional file 1]).

### Treatment effects on kidney function

**Fig. 5:**
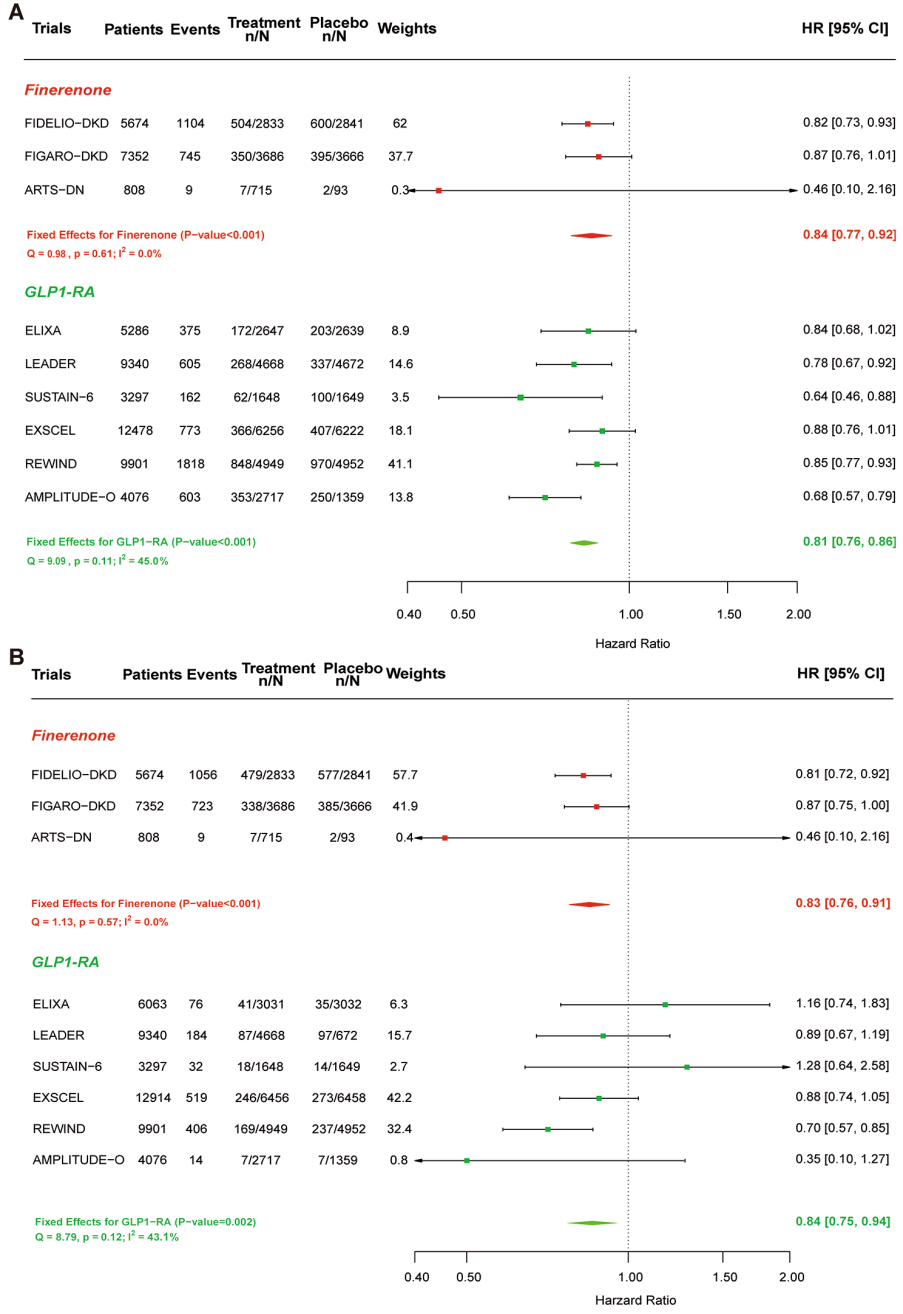
Meta-analysis of finerenone and GLP1-RA trials on renal end points. **(A)** Meta-analysis of finerenone and GLP1-RA trials on a broad kidney end point stratified by drug class. **(B)** Meta-analysis of finerenone and GLP1-RA trials on a narrower kidney outcome excluding macroalbuminuria stratified by drug class

Renal events were not available for the Harmony Outcomes or PIONEER 6 trials. In total, the broad composite kidney endpoint occurred in 6194 patients: 1858 in the finerenone trials and 4336 in the GLP1-RA trials. Finerenone and GLP1-RA are both effective drugs, and GLP1-RA is probably superior to finerenone. GLP1-RA reduced the relative risk of the broad composite kidney outcome significantly by 19% (HR, 0.81; 95% CI, 0.76–0.86; *P* < 0.001; P for heterogeneity, 0.11, I^2^ = 45.0%), whereas there was a 16% reduction with finerenone (HR, 0.84; 95% CI, 0.77–0.92; *P* < 0.001; P for heterogeneity, 0.61, I^2^ = 0.0%; Fig. 5A). Moreover, a previously published meta-analysis has shown that the relative risk reduction of the kidney composite with GLP1-RA appeared to be mainly driven by a reduction in macroalbuminuria [10, 32]. However, with several more recently published trials, our meta-analysis suggests that in addition to reducing the risk of macroalbuminuria, GLP1-RA is comparable to finerenone in reducing the risk of worsening eGFR (Fig. 5B).

## Discussion

Finerenone and GLP1-RA are antidiabetic medications that have been demonstrated to lower the risk of cardiovascular and renal events in patients with T2DM [33, 34]. However, the relative benefits of these drugs between patients with or without pre-existing ASCVD remain uncertain. The present meta-analysis confirms the benefits of finerenone and GLP1-RA in reducing the risk of MACE by approximately 14% in patients with pre-existing ASCVD. In addition, our meta-analysis reveals that these drugs have a significant impact on overall cardiovascular and renal events.

Finerenone is the first non-steroidal mineralocorticoid receptor antagonist that has high affinity and selectivity for the mineralocorticoid receptor (MR) and differs from steroidal mineralocorticoid receptor antagonists (MRAs) and between each other in terms of important physiochemical, pharmacodynamic, and pharmacokinetic properties [35]. Similar to steroidal MRAs, evidence supports a protective role of finerenone in anti-inflammatory, anti-fibrotic and anti-remodeling in both kidney and cardiac tissues, as well as reducing renal and myocardial hypertrophy, BNP, and proteinuria in rodent models, which may be associated with its benefits in renal outcomes [4, 36, 37]. There is evidence from a previous publication that finerenone did not show a significant difference in reduced incidence of MACE based on pre-existing cardiovascular disease status [38]. However, with recently updated trials of finerenone, the present meta-analysis shows that finerenone is likely to reduce the risk of MACE by 15% in patients with established ASCVD (HR, 0.85; 95% CI, 0.78–0.94).

Heart failure is a major cause of hospitalization and is responsible for approximately 7% of cardiovascular deaths, which worsens the prognosis of T2DM patients [39]. Patients with a history of HF at baseline studied in finerenone and GLP1-RA trials have asymptomatic HFrEF (LVEF < 40%) or HFrEF with NYHA class I, HFpEF (LVEF ≥ 50%), or HFmrEF (LVEF 40–49%). Finerenone reduced HHF risk by 22%, whereas there was only a nonsignificant 9% relative risk reduction with GLP1-RA. In addition, a history of HF did not appear to modify the effect of finerenone versus placebo on HHF in the presence of optimized RAS blockade, which might be explained by the special potency of finerenone, such as controlling blood pressure, anti-cardiac remodeling and fibrosis [37]. A newly published meta-analysis has highlighted that the effects of GLP-RA are modified by HF status. Treatment with GLP1-RA did not reduce the risk of HHF in T2DM patients with HF history, particularly HFrEF, but may prevent new-onset HF in T2DM patients without HF [30, 40, 41]. The mechanisms for the different effects of GLP1-RA on HHF prevention among patient with or without HF remains uncertain but, notably, the outcomes showed the reductions in myocardial infarction among the GLP1-RA trials (Figure S3). This finding raises the possibility that GLP1-RA may lower the risk of HHF by reducing the risk of myocardial damage by either reducing body weight, preventing coronary occlusion and myocardial small vessel disease, or ameliorating myocardial muscle damage caused by inflammation or other processes [41, 42]. Meanwhile, GLP1-RA are known to increase heart rate, which may be deleterious to patients with HF. It has reported that GLP1-RA can reduce epicardial fat, which leads to decreased risk of atherosclerotic cardiovascular disease, may explain why GLP1-RA may possibly reduce HHF [43, 44]. Furthermore, the GLP1 receptor, which plays an important role in cardiomyocytes and sinoatrial node cells through a cyclic adenosine monophosphate–dependent pathway, may induce intracellular calcium overload and increase the risk of ventricular ectopy in high-risk patients, such as those with severely depressed left ventricular ejection fraction (LVEF) [30, 40].

With regard to kidney outcomes, the present meta-analysis revealed that GLP1-RA has a similar effect as finerenone on reducing the risk of renal events, even excluding macroalbuminuria, which is in contrast to the findings of another meta-analysis [10]. One of the reasons could be that the REWIND data were not included, because the results were not published at the time of submission. The REWIND data showed the greatest relative risk reductions in renal outcomes among the GLP1-RA trials. Dulaglutide significantly lowered all three components of the composite renal outcome (the development of new macroalbuminuria, a sustained 30% or greater decline in eGFR, or new chronic renal replacement therapy), the largest effect was noted for the development of new macroalbuminuria [27]. The US Food and Drug Administration product label states that liraglutide is at increased risk of renal impairment [45]. Meanwhile, patients with HFrEF and recent hospitalization for acute HF have a greater risk of adverse renal outcomes compared with patients without these conditions. However, a post hoc analysis of FIGHT suggests that liraglutide was not associated with worsening renal function among patients with HFrEF and a recent hospitalization for HF [46]. In addition, the possibility of the benefit of GLP1-RA on renal outcomes will come with the results of the ongoing FLOW (NCT03819153), which specifically tests the kidney benefits of this class [3, 47]. The mechanism underlying the beneficial effects remains unclear but could be, at least in part, due to a combination of GLP-1 receptor agonist-induced weight loss, blood pressure lowering and glycaemia improvements [42]. Interestingly, human GLP-1 analogues such as liraglutide and semaglutide show a favorable risk–benefit profile for MACE and renal outcomes compared with exendin-4-based drugs, including exenatide and lixisenatide. Mechanisms linking the actions of GLP-1 analogues to cardiovascular and renal outcomes are not well understood. It was reported that GLP-1 analogues enhance sodium and water excretion, reduce albumin excretion and have anti-inflammatory effects, which might play a protective role in cardiovascular and renal events. Furthermore, they are resistant to renal elimination due to their large molecular weight or noncovalent binding to albumin, whereas exendin-4 based drugs are eliminated by the kidneys. In addition, exendin-4 analogues are resistant to degradation by dipeptidyl peptidase-4 (DPP-4), which might explain the greater cardiovascular and renal protective effects of GLP-1 analogues than exendin-4 analogues [48, 49].

The major strengths of this meta-analysis are as follows: first, this is the first study to investigate the benefits of finerenone and GLP1-RA between patients with or without established ASCVD. Second, the trials included in this meta-analysis were large, and the statistics were reliable, which provided high-quality evidence to minimize the risk of bias and heterogeneity. Several limitations should be noted in this meta-analysis. We performed this meta-analysis using aggregate trial-level data, and as a result, the observed differences in treatment effects between subgroups were limited. In addition, the included studies were slightly different in the exact inclusion/exclusion criteria and endpoint definitions, which is particularly evident for the definitions of renal outcomes. Randomized trials with head-to-head comparisons would be necessary to demonstrate the possible superiority of a drug class over the other within finerenone and GLP1-RA. Third, there were differences in drug doses, patient characteristics, and background therapy. Finally, the present meta-analysis is not able to assess potential incremental or additive treatment effects when both drug classes are used in combination.

## Conclusion

In conclusion, finerenone and GLP1-RA lead to a risk reduction in MACE to a similar extent in patients with established ASCVD. In patients with T2DM, finerenone has significant effects on reducing the risk of progression of kidney disease and HHF, whereas GLP1-RA is associated with a lower risk of renal events but has no effect on HHF. Therefore, the prevention of HHF potentially by finerenone over GLP1-RA should be considered for decision makers in adults with T2DM.

### Electronic supplementary material

Below is the link to the electronic supplementary material.

Supplementary Material 1

Supplementary Material 2

## Data Availability

All data generated or analyzed during this study are included in this published article.

## Abbreviations

GLP1-RA: Glucagon-like peptide 1 receptor agonists
T2DM: Type 2 diabetes mellitus
MACE: Major adverse cardiovascular events
HHF: Hospitalization for heart failure
HR: Hazard ratio
CI: Confidence interval
ESRD: End-stage renal disease
CKD: Chronic kidney disease
eGFR: Estimated glomerular filtration rate
RCTs: Randomized control trials
ADA: American Diabetes Association
ASCVD: Atherosclerotic cardiovascular disease
PRISMA-P: Preferred reporting items for systematic reviews and meta-analyses protocols
T1DM: Type 1 diabetes mellitus
MA: Mineralocorticoid receptor
MRA: mineralocorticoid receptor antagonist
CVD: Cardiovascular disease
HFrEF: Heart failure with reduced ejection fraction
HFpEF: Heart failure with preserved ejection fraction
LVEF: left ventricular ejection fraction
HFmrEF: Heart failure with mildly reduced ejection fraction
DPP-4: Dipeptidyl peptidase-4
