# Supplementary material for: Effects of finerenone and glucagon-like peptide 1 receptor agonists on cardiovascular and renal outcomes in type 2 diabetes mellitus: a systematic review and meta-analysis
Source: Diabetol Metab Syndr. 2024 Jan 11;16:14. doi: 10.1186/s13098-023-01251-2 (PMC10782753; doi:10.1186/s13098-023-01251-2)

Search Algorithm

PubMed:

(“Diabetes Mellitus, Type 2”[Mesh] OR “diabetes mellitus type 2”[tiab] OR “type 2 diabetes”[tiab] OR“T2D*”[tiab]) AND (“finerenone"[tiab] OR “Glucagon-Like Peptide-1 receptor”[MeSH] OR “GLP-1”[tiab] OR “GLP1 receptor agonist”[tiab] OR “glucagon-like peptide-1 receptor agonist”[tiab] OR “exenatide”[MeSH] OR “liraglutide”[MeSH] OR “lixisenatide”[tiab] OR “albiglutide”[tiab] OR “dulaglutide”[tiab] OR “semaglutide”[tiab]) AND (random*[tw] OR "Letter"[pt] OR “trial”[tiab]) AND (“Myocardial Infarction”[Mesh] OR “Myocardial Infarction” [tiab] OR “stroke”[Mesh] OR “stroke”[tiab] OR “death”[Mesh] OR “death”[tiab] OR “MACE”[tiab] OR “major adverse cardiovascular events”[tiab] OR “major adverse cardiac events”[tiab] OR “heart failure”[Mesh] OR “heart failure”[tiab])

Embase:

('non insulin dependent diabetes mellitus'/exp OR T2DM:ab,ti OR Diabetes Mellitus, Type 2:ab,ti OR diabetes mellitus type 2:ab,ti OR type 2 diabetes:ab,ti OR T2D:ab,ti) AND (finerenone:ab,ti OR Glucagon-Like Peptide-1 receptor:ab,ti OR GLP-1:ab,ti OR GLP1 receptor agonist:ab,ti OR glucagon-like peptide-1 receptor agonist:ab,ti OR lixisenatide:ab,ti OR liraglutide:ab,ti OR semaglutide:ab,ti OR exenatide:ab,ti OR albiglutide:ab,ti OR dulaglutide:ab,ti) AND (random*:ti,ab,de AND placebo:ab,ti) AND 'controlled study'/de AND **(**'heart infarction'/exp OR 'myocardial infarction':ab,ti OR 'cerebrovascular accident'/exp OR 'stroke':ab,ti OR 'death'/exp OR 'death':ab,ti OR 'major adverse cardiac event'/exp OR 'MACE':ab,ti OR 'major adverse cardiovascular event':ab,ti OR 'heart failure'/exp OR 'heart failure':ab,ti**)**

Cochrane Library:

#1 MeSH descriptor: [Diabetes Mellitus] this term only 11675

#2 (Diabetes Mellitus type 2):ti,ab,kw OR (type 2 Diabetes Mellitus):ti,ab,kw OR (Type 2 Diabetes):ti,ab,kw OR (Diabetes, Type 2):ti,ab,kw OR (Diabetes Mellitus, Type II):ti,ab,kw 56198

#3 (Noninsulin Dependent Diabetes Mellitus):ti,ab,kw 637

#4 MeSH descriptor: [Glucagon-Like Peptide-1 Receptor] this term only 306

#5 (GLP1):ti,ab,kw OR (GLP1 receptor agonist):ti,ab,kw OR (glucagon-like peptide-1 receptor agonist):ti,ab,kw OR (Lixisenatide):ti,ab,kw OR (Albiglutide):ti,ab,kw 1454

#6 (Dulaglutide):ti,ab,kw OR (Semaglutide):ti,ab,kw OR (Finerenone):ti,ab,kw 1392

#7 MeSH descriptor: [Exenatide] this term only 621

#8 MeSH descriptor: [Liraglutide] this term only 899

#9 (Randomized controlled study):ti,ab,kw OR (RCT):ti,ab,kw 677969

#10 (heart infarction):ti,ab,kw OR (myocardial infarction):ti,ab,kw OR (cerebrovascular accident):ti,ab,kw OR (stroke):ti,ab,kw OR (death):ti,ab,kw 150670

#11 (major adverse cardiac event):ti,ab,kw OR (MACE):ti,ab,kw OR (major adverse cardiovascular event):ti,ab,kw OR (heart failure):ti,ab,kw 47962

#12 (#1 OR #2 OR #3) AND (#4 OR #5 OR #6 OR #7 OR #8) AND #9 AND (#10 OR #11) with Publication Year from 2010 to 2022, in Trials 228

Table S1. Definition of Cardiovascular Disease

Trial	Definition of Cardiovascular Disease
Finerenone vs placebo	
**FIDELIO-DKD**	**1.Coronary Artery Disease:**<br>Previous Myocardial Infarction (MI)<br>History of coronary revascularization (percutaneous coronary intervention or coronary artery bypass graft)<br>Angiographically proven stenosis ≥ 50% in at least one major epicardial coronary artery<br>**2.Cerebrovascular Disease:**<br>Previous ischemic stroke [transient ischemic attack (TIA) alone not sufficient to fulfill this criterion]<br>**3.Peripheral Arterial Disease:**<br>Previous non-traumatic leg amputation<br>History of lower-limb revascularization (either surgical or percutaneous)<br>History of intermittent claudication with ankle brachial pressure index (ABPI) of ≤ 0.80 in at least one side Previous carotid endarterectomy or carotid stenting
**FIGARO-DKD**	**1.Coronary Artery Disease:**<br>Previous Myocardial Infarction (MI)<br>History of coronary revascularization (percutaneous coronary intervention or coronary artery bypass graft)<br>Angiographically proven stenosis ≥ 50% in at least one major epicardial coronary artery<br>**2.Cerebrovascular Disease:**<br>Previous ischemic stroke [transient ischemic attack (TIA) alone not sufficient to fulfill this criterion]<br>**3.Peripheral Arterial Disease:**<br>Previous non-traumatic leg amputation<br>History of lower-limb revascularization (either surgical or percutaneous)<br>History of intermittent claudication with ankle brachial pressure index (ABPI) of ≤ 0.80 in at least one side Previous carotid endarterectomy or carotid stenting
**ARTS-DN**	Not included

GLP-1RA vs placebo	
**ELIXA**	Myocardial infarction or hospitalized for unstable angina within the previous 180 days before enrollment.
**LEADER**	**Age ≥50 and ≥1 of the following criteria:**<br>1. Prior MI<br>2. Prior stroke or TIA<br>3. Prior coronary, carotid or peripheral arterial revascularization<br>4. >50% stenosis of coronary, carotid, or lower extremity arteries<br>5. History of symptomatic CHD documented by positive exercise stress test or any cardiac imaging or unstable angina with ECG changes<br>6. Asymptomatic cardiac ischemia documented by positive nuclear imaging test, exercise test or dobutamine stress echo<br>7. Chronic heart failure NYHA class II-III<br>8. Chronic kidney failure (eGFR <60 ml/min/1.73m2)
**SUSTAIN-6**	**Age ≥50 and ≥1 of the following criteria:**<br>1. Prior MI<br>2. Prior stroke or TIA<br>3. Prior coronary, carotid or peripheral arterial revascularization<br>4. >50% stenosis on angiography or imaging of coronary, carotid or lower extremities arteries<br>5. History of symptomatic coronary heart disease documented by e.g. positive exercise stress test or any cardiac imaging or unstable angina with ECG changes<br>6. Asymptomatic cardiac ischemia documented by positive nuclear imaging test or exercise test or stress echo or any cardiac imaging<br>7. Chronic heart failure New York Heart Association (NYHA) class II-III<br>8. Chronic kidney impairment (eGFR <60 ml/min/1.73 m^2 per MDRD)

**EXSCEL**	**Prior Cardiovascular event defined as:**<br>1. History of a major clinical manifestation of coronary artery disease i.e. myocardial infarction, surgical or percutaneous (balloon and/or stent) coronary revascularization procedure, or coronary angiography showing at least one stenosis ⩾50% in a major epicardial artery or branch vessel<br>2. Ischemic cerebrovascular disease, including: History of ischemic stroke; strokes not known to be hemorrhagic will be allowed as part of this criterion; transient ischemic attacks (TIAs) are not included<br>3. History of carotid arterial disease as documented by ⩾50% stenosis documented by carotid ultrasound, magnetic resonance imaging (MRI), or angiography, with or without symptoms of neurologic deficit<br>4. Atherosclerotic peripheral arterial disease, as documented by objective evidence such as amputation due to vascular disease, current symptoms of intermittent claudication confirmed by an ankle-brachial pressure index or toe-brachial pressure index less than 0.9, or history of surgical or percutaneous revascularization procedure.
**HARMONY**	**Established cardiovascular disease, including at least 1 of the following:**<br>**Coronary artery disease with either of the following:**<br>a. Documented history of spontaneous myocardial infarction, at least 30 days prior to Screening.<br>b. Documented coronary artery disease (CAD) ⩾ 50% stenosis in 1 or more<br>c. Major epicardial coronary arteries, determined by invasive angiography, or history of surgical or percutaneous (balloon and/or stent) coronary revascularization procedure (at least 30 days prior to Screening for percutaneous procedures and at least 5 years prior to Screening for coronary artery bypass graft.)<br>**Cerebrovascular disease – any of the following:**<br>a. Documented history of ischaemic stroke, at least 90 days prior to study entry.<br>b. Carotid arterial disease with 50% stenosis documented by carotid ultrasound, magnetic resonance imaging or angiography, with or without symptoms of neurologic deficit.<br>c. Carotid vascular procedure (e.g. stenting or surgical revascularisation), at least 30 days prior to Screening.<br>**Peripheral arterial disease (PAD) with either of the following:**<br>a. intermittent claudication and ankle: brachial index < 0.9 in at least one ankle

	b. prior non-traumatic amputation, or peripheral vascular procedure (e.g. stenting or surgical revascularisation), due to peripheral arterial ischaemia.
**REWIND**	**If age ≥50 years and established clinical vascular disease defined as 1 or more of the following:**<br>1. History of MI<br>2. History of ischemic stroke<br>3. History of coronary, carotid, or peripheral artery revascularization. If prior coronary artery bypass grafting (CABG), the CABG should have been performed >2 years prior to randomization. If prior carotid or peripheral artery revascularization, the revascularization should have been performed >2 months prior to randomization.<br>4. Hospitalization for unstable angina with ECG changes (new or worsening ST or T wave changes), or myocardial ischemia on imaging, or need for percutaneous coronary intervention (PCI)<br>**If age ≥55 years and subclinical vascular disease defined as 1 or more of the following:**<br>1. History of myocardial ischemia by a stress test or with cardiac imaging, with or without history of exertional angina<br>2. >50% vascular stenosis with imaging of the coronary, carotid, or lower extremity arteries, with or without claudication history<br>3. Ankle-brachial index <0.9<br>4. 2 consecutive values or a documented history of persistent eGFR<60 mL/minute/$1.73m^2$<br>5. History of hypertension with documented LV hypertrophy on an ECG or echocardiogram<br>6. Documented history of persistent microalbuminuria, or macroalbuminuria; or 2 consecutive urine samples demonstrating micro- or macroalbuminuria.

**AMPLITUDE-O**	**Participants who meet at least one of the 8 CVD criteria of the following:**<br>**Presence of Coronary Artery Disease (CAD):**<br>1. Documented (with ECG changes and cardiac enzymes) history of symptomatic myocardial infarction (>2 months prior to screening)<br>2. Evidence of multi-vessel coronary artery disease, ie, ≥2 major coronary arteries or the left main coronary artery, documented by any of the following:<br>- Presence of a significant stenosis (imaging evidence of ≥50% narrowing of the luminal diameter measured during a coronary angiography or a multi-sliced computed tomography)<br>- Previous revascularization of ≥2 major coronary arteries (percutaneous transluminal coronary angioplasty (with or without stent), or CABG) (>2 months prior to screening)<br>- The combination of revascularization in one major coronary artery (>2 months prior to screening) and the presence of a significant stenosis in another major coronary artery {≥50% luminal narrowing during an angiography (coronary or multi-sliced computed tomography)}<br>3. Evidence of single vessel coronary artery disease of at least ≥50% luminal narrowing during a angiography (coronary or multi-sliced computed tomography) not subsequently successfully revascularized, with at least 1 of the following:<br>- A positive non-invasive stress test for ischemia<br>- Hospitalization for unstable angina within the prior 12 months<br>**Presence of Cerebrovascular Disease**<br>4. History of Ischemic or Haemorrhagic stroke (>2 months prior to screening)<br>**Presence of Peripheral Arterial Disease (PAD) (symptomatic or not)**<br>5. Previous limb angioplasty<br>6. Stenting or bypass surgery of peripheral artery<br>7. Previous limb or foot amputation due to circulatory insufficiency<br>8. Ankle-brachial index <0.9<br>9. Angiographic evidence of PAD

**PIONEER 6**	**Age ≥50 years at screening and at least one of the following conditions:**<br>1. Prior myocardial infarction<br>2. Prior stroke or transient ischemic attack<br>3. Prior coronary, carotid, or peripheral arterial revascularization<br>4. >50% stenosis on angiography or imaging of coronary, carotid, or lower extremity arteries<br>5. History of symptomatic coronary heart disease documented by e.g., positive exercise stress test or any cardiac imaging or unstable angina pectoris with electrocardiogram changes<br>6. Asymptomatic cardiac ischemia documented by positive nuclear imaging test or exercise test or stress echo or any cardiac imaging<br>7. Chronic heart failure New York Heart Association (NYHA) class 2–3<br>8. Moderate renal impairment (estimated glomerular filtration rate [eGFR] 30 to 59 ml/min/1.73 m^2).

Table S2. The definition of the broad kidney endpoint of the included trials.

Trial	Definition of the Broad Kidney Endpoint
Finerenone vs placebo	
FIDELIO-DKD[1]	Onset of kidney failure, a sustained decrease of eGFR ≥40% from baseline over at least 4 Weeks, or renal death.
FIGARO-DKD[2]	Onset of kidney failure, a sustained decrease of eGFR ≥40% from baseline over at least 4 Weeks, or renal death.
ARTS-DN[3]	Ratio of UACR at Day 90 to UACR at Baseline
GLP1-RA vs placebo	
ELIXA[4]	New-onset macroalbuminuria.
LEADER[5]	New-onset persistent macroalbuminuria, persistent doubling of the serum creatinine level and an eGFR <45 ml/min/1.73m^2, the need for continuous renal-replacement therapy with no reversible cause of the kidney disease, or death from kidney disease.
SUSTAIN-6[6]	Persistent macroalbuminuria, persistent doubling of the serum creatinine and a creatinine clearance <45 ml/min/1.73m^2 (using the MDRD equation), or the need for continuous renal-replacement therapy.
EXSCEL[7]	New-onset macroalbuminuria, 40% eGFR decline, the need for renal replacement therapy, and death from kidney disease.
HARMONY	No outcomes reported.
REWIND[8]	The development of macroalbuminuria, a sustained 30% or greater decline in eGFR, or new chronic renal replacement therapy comprising dialysis or renal transplantation.
AMPLITUDE-O[9]	Incident macroalbuminuria, plus an increase in the urinary albumin-to-creatinine ratio of ≥30% from baseline, a sustained decrease in the eGFR of ≥40% for ≥30 days, renal-replacement therapy for ≥90 days, or a sustained eGFR of <15 ml per minute per 1.73 m^2 for ≥30 days.
PIONEER 6	No outcomes reported.

Supplementary Reference:

[1] Effect of Finerenone on Chronic Kidney Disease Outcomes in Type 2 Diabetes," New England journal of medicine, Vol.383, no.23, pp.2219-2229, 2020.

[2] Cardiovascular Events with Finerenone in Kidney Disease and Type 2 Diabetes," New England journal of medicine, Vol.385, no.24, pp.2252-2263, 2021.

[3] Effect of Finerenone on Albuminuria in Patients with Diabetic Nephropathy: A Randomized Clinical Trial," JAMA, Vol.314, no.9, pp.884-94, 2015.

[4] Lixisenatide and renal outcomes in patients with type 2 diabetes and acute coronary syndrome: an exploratory analysis of the ELIXA randomised, placebo-controlled trial. Lancet Diabetes Endocrinol. 2018; 6:859-869.

[5] Liraglutide and Renal Outcomes in Type 2 Diabetes. N Engl J Med. 2017; 377:839-848.

[6] Semaglutide and Cardiovascular Outcomes in Patients with Type 2 Diabetes. N Engl J Med. 2016; 375:1834-1844.

[7] Renal Outcomes in the EXenatide Study of Cardiovascular Event Lowering (EXSCEL). Diabetes. 2018; 67:522-P.

[8] Dulaglutide and renal outcomes in type 2 diabetes: an exploratory analysis of the REWIND randomised, placebo-controlled trial," Lancet (london, england), Vol.394, no.10193, pp.131-138, 2019.

[9] Cardiovascular and renal outcomes with efpeglenatide in type 2 diabetes," New England Journal of Medicine, Vol.385, no.10, pp.896-907, 2021.

Figure S1. Meta-Analysis of GLP1-RA trials stratified by renal function for the composite of myocardial infarction, stroke, and cardiovascular death (MACE)

Trials	Patients	Events	Treatment n/N	Placebo n/N	Weights	HR [95% CI]
**Baseline eGFR ≥ 60 mL/min/1.73m²**						
LEADER	7182	907	436/3552	471/3630	22.5	0.95 [0.84, 1.07]
SUSTAIN−6	2358	156	63/1179	93/1179	17.9	0.68 [0.50, 0.92]
EXSCEL	11514	1169	549/5769	620/5745	22.7	0.88 [0.79, 0.98]
HARMONY	7241	522	222/4417	300/2824	21.6	0.47 [0.40, 0.56]
PIONEER 6	2308	92	41/1150	51/1158	15.3	0.81 [0.54, 1.21]
Random Effects for GLP1−RA (P−value=0.024)<br>Q = 49.46, p = 0.00; I^2 = 91.2%						0.74 [0.57, 0.96]
**Baseline eGFR < 60 mL/min/1.73m²**						
LEADER	2158	395	172/1116	223/1042	27.2	0.72 [0.60, 0.86]
SUSTAIN−6	939	98	45/469	53/470	13.3	0.85 [0.58, 1.24]
EXSCEL	3191	567	283/1565	284/1626	30.2	1.04 [0.89, 1.20]
HARMONY	2222	244	116/1098	128/1124	22.2	0.93 [0.73, 1.18]
PIONEER 6	856	45	20/434	25/422	7.1	0.78 [0.44, 1.38]
Random Effects for GLP1−RA (P−value=0.117)<br>Q = 9.64, p = 0.05; I^2 = 56.2%						0.87 [0.74, 1.03]

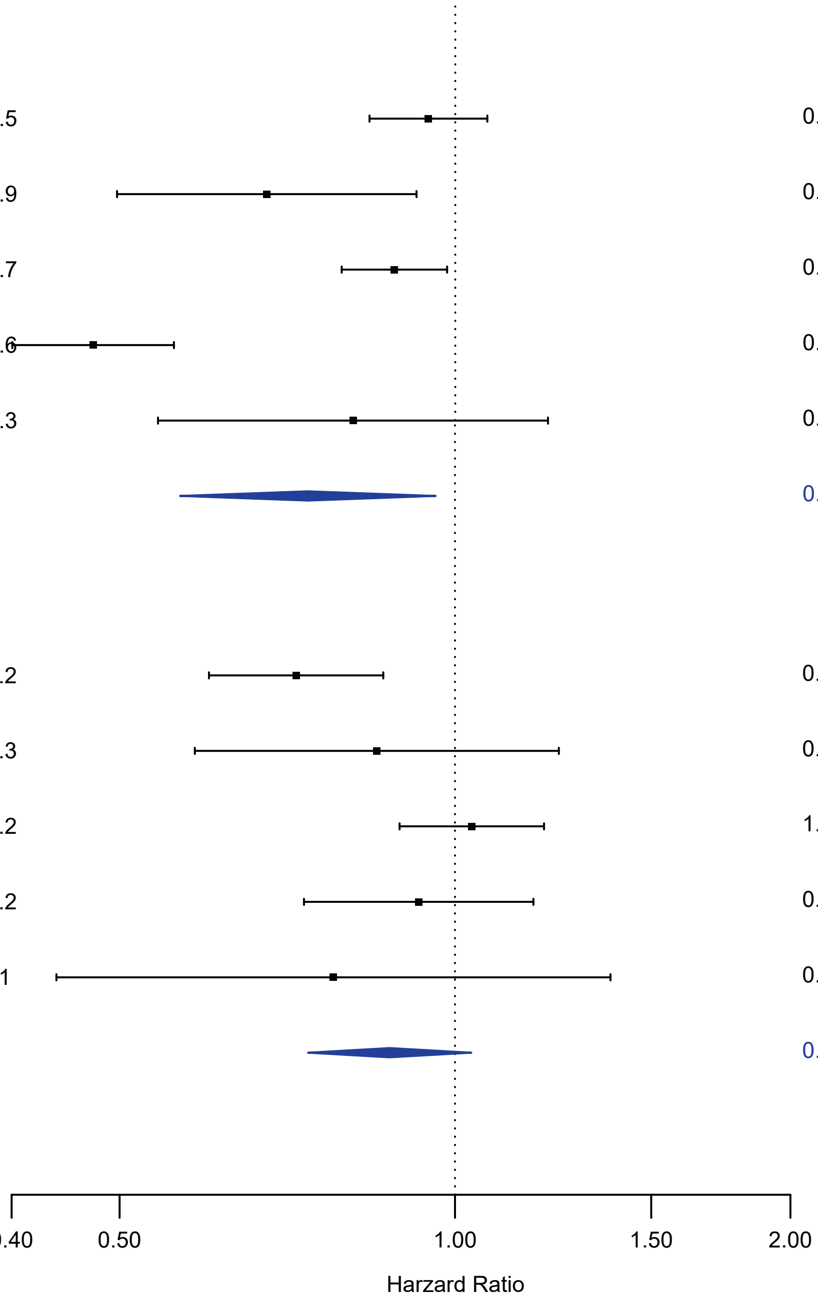

Figure S2. Meta-Analysis of finerenone and GLP1-RA trials stratified by presence of established atherosclerotic cardiovascular disease for the composite of myocardial infarction, stroke, and cardiovascular death (MACE)

Trials	Patients	Events	Treatment n/N	Placebo n/N	Weights	HR [95% CI]
Established atherosclerotic cardiovascular disease						
Finerenone						
FIDELIO−DKD	2605	494	231/1303	263/1302	43.2	0.85 [0.71, 1.02]
FIGARO−DKD	3330	612	280/1676	332/1654	56.8	0.82 [0.70, 0.96]
**Fixed Effects for Finerenone (P−value=0.003)**<br>**Q = 0.09 , p = 0.77 ; I² = 0.0 %**						**0.85 [0.77, 0.90]**
GLP1-RA						
ELIXA	6068	805	406/3034	399/3034	14.7	1.02 [0.89, 1.17]
LEADER	7598	1165	536/3831	629/3767	21.1	0.83 [0.74, 0.93]
SUSTAIN−6	2735	235	98/1353	137/1382	4.0	0.72 [0.55, 0.93]
EXSCEL	10782	1508	722/5394	786/5388	28.0	0.90 [0.82, 1.00]
HARMONY	9463	766	338/4731	428/4732	14.0	0.78 [0.68, 0.90]
REWIND	3114	595	280/1560	315/1554	10.7	0.87 [0.74, 1.02]
AMPLITUDE−O	3650	299	177/2420	122/1230	5.3	0.71 [0.57, 0.90]
PIONEER 6	2695	125	57/1350	68/1345	2.2	0.83 [0.58, 1.17]
**Fixed Effects for GLP1−RA (P−value<0.001)**<br>**Q = 13.54 , p = 0.06 ; I² = 48.3 %**						**0.86 [0.82, 0.90]**
Without established atherosclerotic cardiovascular disease						
Finerenone						
FIDELIO−DKD	3069	293	136/1530	157/1539	44.5	0.87 [0.70, 1.08]
FIGARO−DKD	4022	365	178/2010	187/2012	55.5	0.95 [0.78, 1.16]
**Fixed Effects for Finerenone (P−value=0.24)**<br>**Q = 0.36, p = 0.55;I² = 0.0%**						**0.92 [0.79, 1.06]**
GLP1-RA						
LEADER	1742	137	72/837	65/905	13.6	1.20 [0.87, 1.65]
SUSTAIN-6	562	19	10/295	9/267	1.8	1.01 [0.41, 2.44]
EXSCEL	3970	236	117/1962	119/2008	23.0	1.01 [0.79, 1.29]
REWIND	6221	594	277/3093	317/3128	59.8	0.88 [0.76, 1.03]
AMPLITUDE-O	426	15	12/297	3/129	0.9	1.74 [0.50, 6.05]
PIONEER 6	488	12	4/241	8/247	1.0	0.51 [0.16, 1.68]
**Fixed Effects for Finerenone (P−value=0.41)**<br>**Q = 5.00, p = 0.42;I² = 0.1%**						**0.94 [0.83, 1.06]**

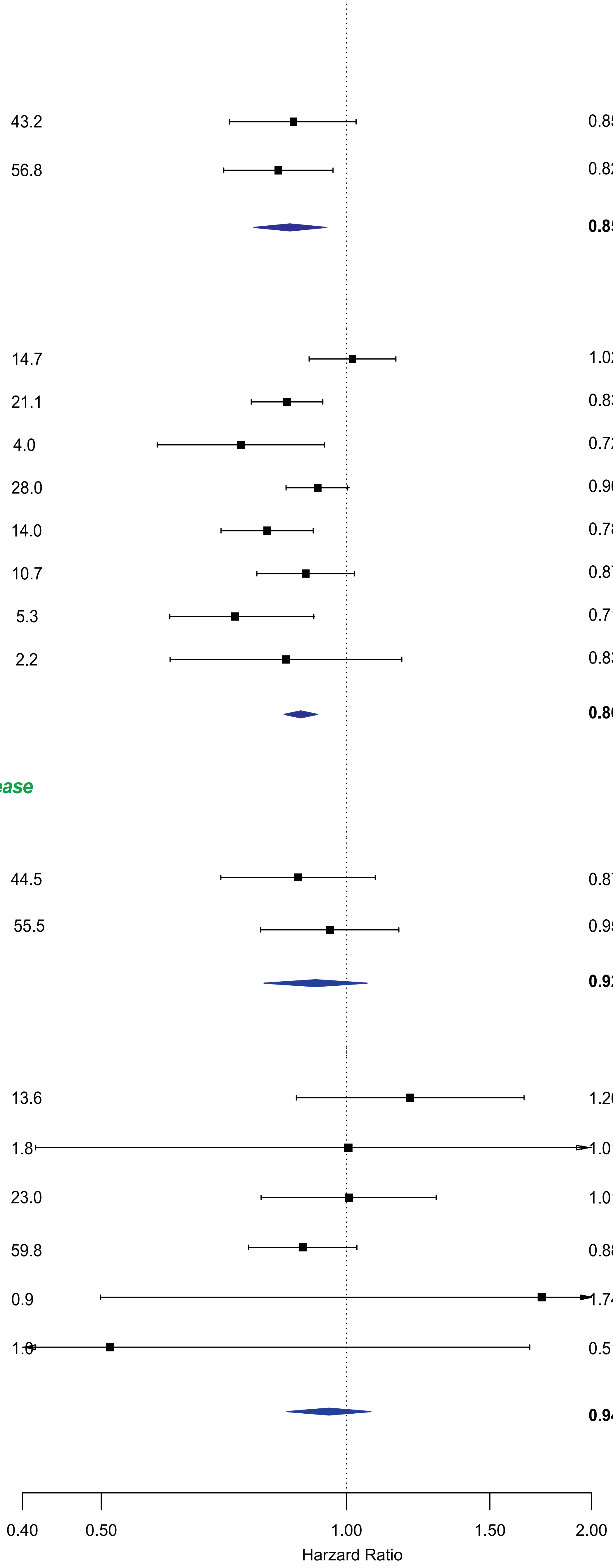

Figure S3. Meta-Analysis of finerenone and GLP1-RA trials on myocardial infarction stratified by drug class

Trials	Patients	Events	Treatment Events per 100 ptyrs	Placebo Events per 100 ptyrs	Weights	HR [95% CI]
Finerenone						
FIDELIO−DKD	5674	157	0.94	1.17	42.7	0.80 [0.58, 1.09]
FIGARO−DKD	7352	205	0.85	0.85	57.3	0.99 [0.76, 1.31]
**Fixed Effects for Myocardial Infarction (P−value=0.34)**<br>**Q = 1.00, p = 0.32; I^2 = 0.5%**						**0.90 [0.74, 1.11]**
GLP1-RA						
ELIXA	6068	531	4.2	4.1	16.6	1.03 [0.87, 1.22]
LEADER	9340	631	1.6	1.9	19.2	0.86 [0.73, 1.00]
SUSTAIN−6	3297	111	1.4	1.92	3.4	0.74 [0.51, 1.08]
EXSCEL	14752	976	2.1	2.1	28.5	0.97 [0.85, 1.10]
HARMONY	9463	421	2.43	3.26	12.5	0.75 [0.61, 0.90]
REWIND	9901	454	0.87	0.91	13.5	0.96 [0.79, 1.15]
AMPLITUDE−O	4076	149	1.9	2.4	4.3	0.75 [0.54, 1.05]
PIONEER 6	3183	68	1.8	1.5	2.1	1.18 [0.73, 1.90]
**Fixed Effects for Myocardial Infarction (P−value=0.008)**<br>**Q = 11.21, p = 0.13; I^2 = 37.6%**						**0.91 [0.85, 0.98]**

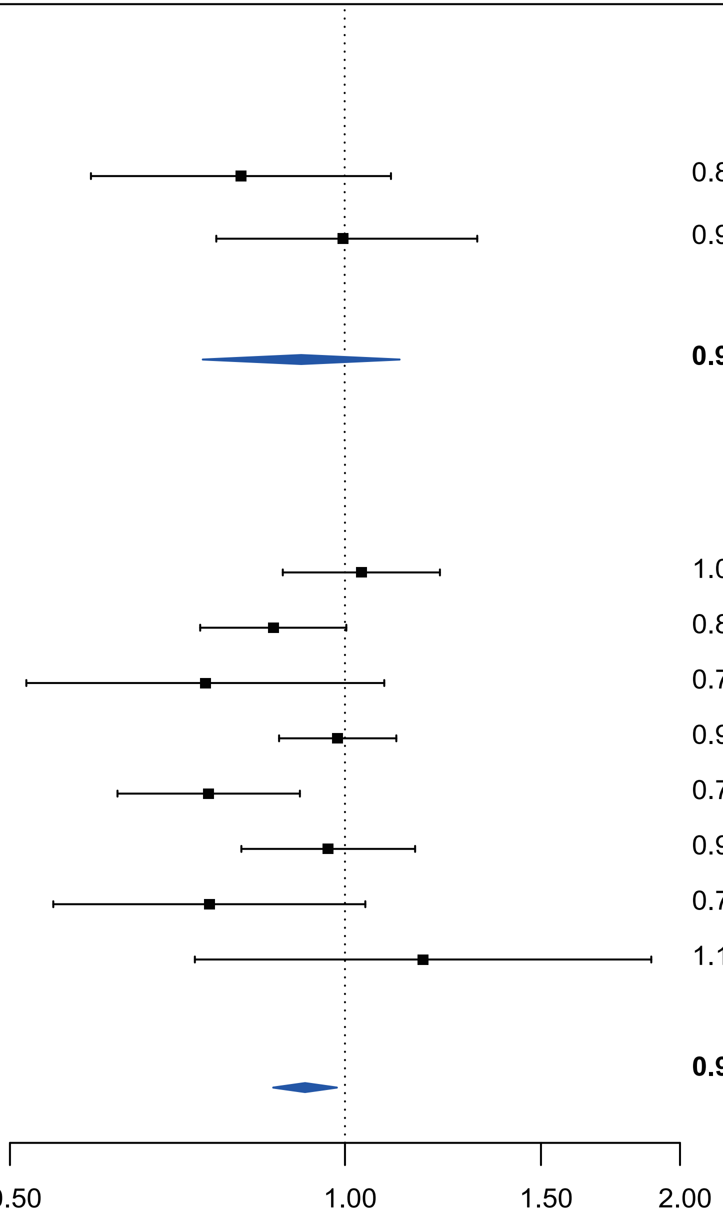

P value for subgroup differences between the 2 drug classes: 0.94

Figure S4. Meta-Analysis of finerenone and GLP1-RA trials on stroke stratified by drug class

Trials	Patients	Events	Treatment Events per 100 ptyrs	Placebo Events per 100 ptyrs	Weights	HR [95% CI]
Finerenone						
FIDELIO−DKD	5674	177	1.21	1.18	44.3	1.03 [0.76, 1.38]
FIGARO−DKD	7352	219	0.89	0.92	55.7	0.97 [0.74, 1.26]
**Fixed Effects for Stroke (P−value=0.97)**<br>**Q = 0.09, p = 0.77; I^2 = 0.0%**						**1.00 [0.82, 1.21]**
GLP1-RA						
ELIXA	6068	127	1	0.9	7.7	1.12 [0.79, 1.58]
LEADER	9340	372	1	1.1	23.2	0.86 [0.71, 1.06]
SUSTAIN−6	3297	71	0.8	1.31	4.1	0.61 [0.38, 0.99]
EXSCEL	14752	405	0.8	0.9	24.9	0.85 [0.70, 1.03]
HARMONY	9463	202	1.25	1.45	12.5	0.86 [0.66, 1.14]
REWIND	9901	363	0.61	0.81	21.5	0.76 [0.62, 0.94]
AMPLITUDE−O	4076	78	1	1.3	4.5	0.74 [0.47, 1.17]
PIONEER 6	3183	28	0.6	0.8	1.7	0.74 [0.35, 1.57]
**Fixed Effects for Stroke (P−value<0.001)**<br>**Q = 5.72, p = 0.57; I^2 = 0.0%**						**0.83 [0.76, 0.92]**

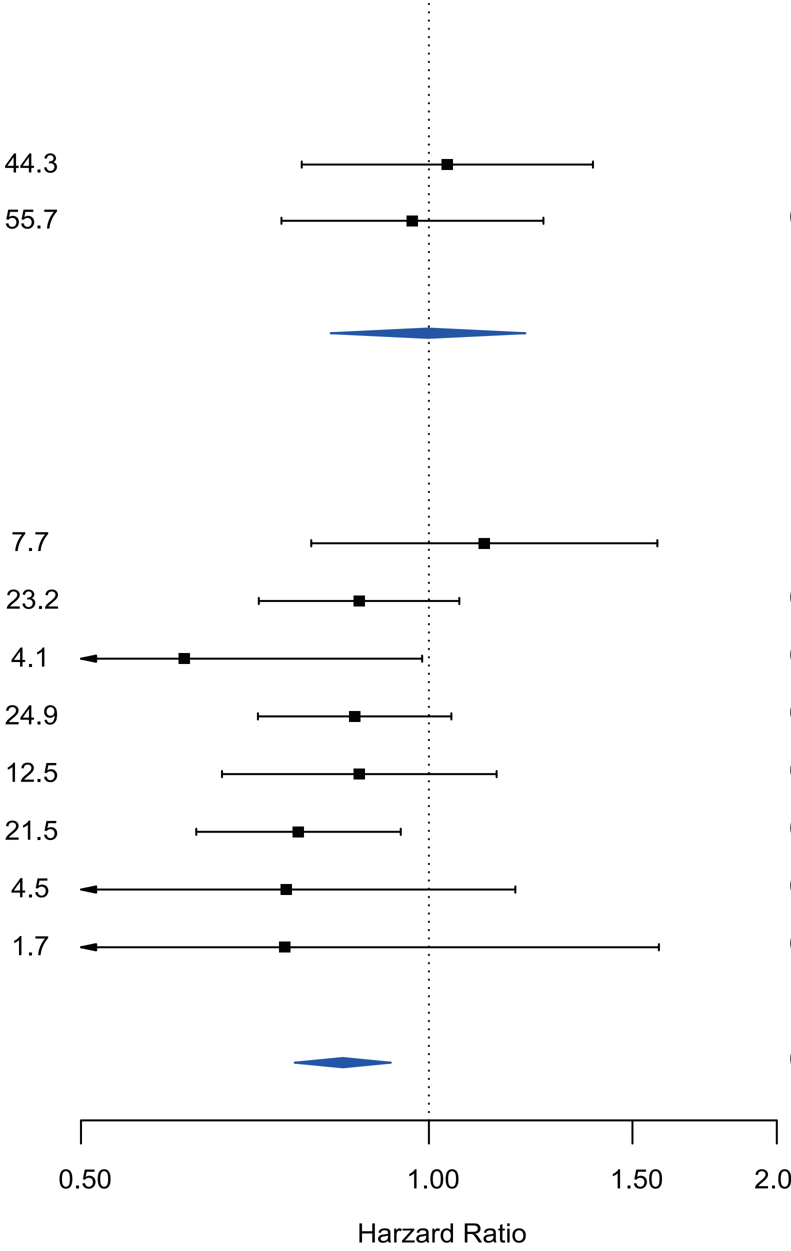

P value for subgroup differences between the 2 drug classes: 0.11

Figure S5. Meta-Analysis of finerenone and GLP1-RA trials on cardiovascular death stratified by drug class

Trials	Patients	Events	Treatment Events per 100 ptyrs	Placebo Events per 100 ptyrs	Weights	HR [95% CI]
Finerenone						
FIDELIO−DKD	5674	278	1.69	1.99	41.2	0.86 [0.68, 1.08]
FIGARO−DKD	7352	408	1.56	1.74	58.8	0.90 [0.74, 1.09]
**Fixed Effects for CV Death (P−value=0.10)**<br>**Q = 0.09, p = 0.77; I^2 = 0.0%**						**0.88 [0.76, 1.02]**
GLP1-RA						
ELIXA	6068	314	2.3	2.4	11.4	0.98 [0.78, 1.22]
LEADER	9340	497	1.2	1.6	19.4	0.78 [0.66, 0.93]
SUSTAIN−6	3297	90	1.29	1.35	3.4	0.98 [0.65, 1.48]
EXSCEL	14752	723	1.4	1.5	26.3	0.88 [0.76, 1.02]
HARMONY	9463	252	1.61	1.72	9.5	0.93 [0.73, 1.19]
REWIND	9901	663	1.22	1.34	24.2	0.91 [0.78, 1.06]
AMPLITUDE−O	4076	125	1.5	2.1	4.4	0.72 [0.50, 1.03]
PIONEER 6	3183	45	0.7	1.4	1.5	0.49 [0.27, 0.92]
**Fixed Effects for CV Death (P−value<0.001)**<br>**Q = 8.03, p = 0.33; I^2 = 12.8%**						**0.87 [0.81, 0.94]**

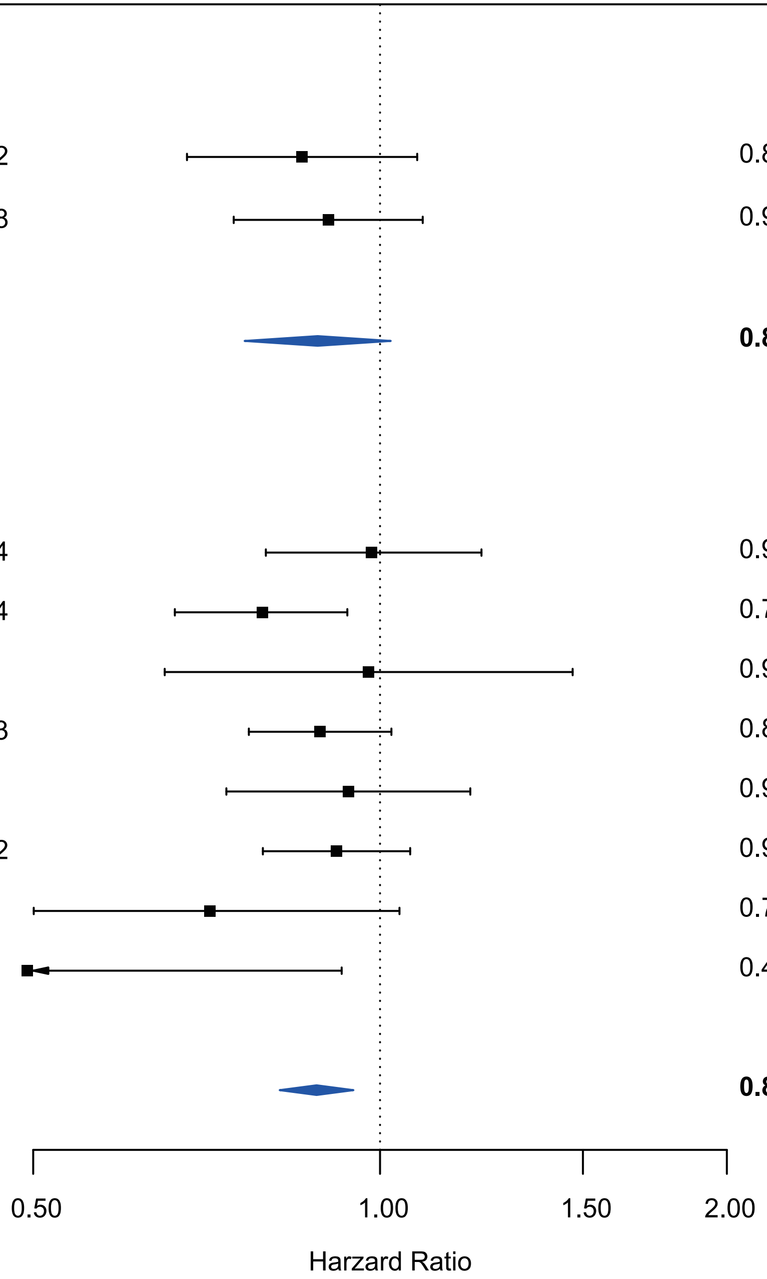

P value for subgroup differences between the 2 drug classes: 0.86

Figure S6. Treatment effects of finerenone according to HF history status.

A. HF hospitalization.

Trials	Patients	Events	Treatment n/N	Placebo n/N	Weights	HR [95% CI]
**No HF**						
FIDELIO−DKD	5238	237	116/2638	121/2600	53.1	0.95 [0.73, 1.22]
FIGARO−DKD	6781	209	88/3396	121/3385	46.9	0.72 [0.55, 0.95]
Fixed Effects for finerenone (P−value=0.058)						0.83 [0.69, 1.00]
Q = 2.00, p = 0.16;I² = 49.9%						
**HF**						
FIDELIO−DKD	436	64	23/195	41/241	47.8	0.65 [0.39, 1.09]
FIGARO−DKD	571	71	29/290	42/281	52.2	0.70 [0.43, 1.15]
Fixed Effects for finerenone (P−value=0.036)						0.68 [0.47, 0.96]
Q = 0.01, p = 0.91; I² = 0.0%						

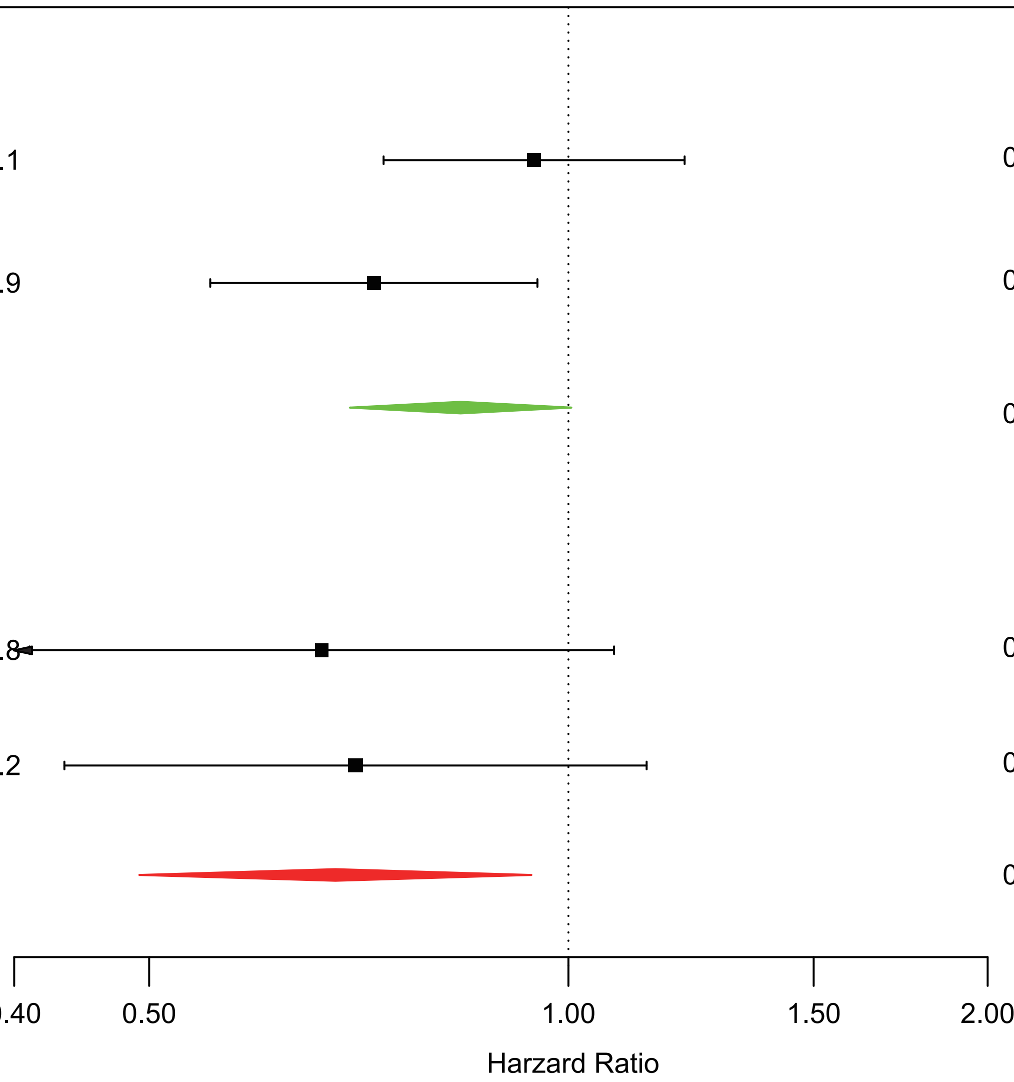

B. Composite of HF hospitalization or cardiovascular death.

Trials	Patients	Events	Treatment n/N	Placebo n/N	Weights	HR [95% CI]
**No HF**						
FIDELIO−DKD	5238	442	210/2638	232/2600	45.4	0.89 [0.74, 1.07]
FIGARO−DKD	6781	519	236/3396	283/3385	54.6	0.83 [0.70, 0.98]
Fixed Effects for finerenone (P−value=0.015)						0.86 [0.76, 0.97]
Q = 0.32, p = 0.57; I² = 0.0%						
**HF**						
FIDELIO−DKD	436	96	39/195	57/241	45.3	0.80 [0.53, 1.20]
FIGARO−DKD	571	122	54/290	68/281	54.7	0.84 [0.58, 1.22]
Fixed Effects for finerenone (P−value=0.162)						0.82 [0.62, 1.08]
Q = 0.03, p = 0.86; I² = 0.0%						

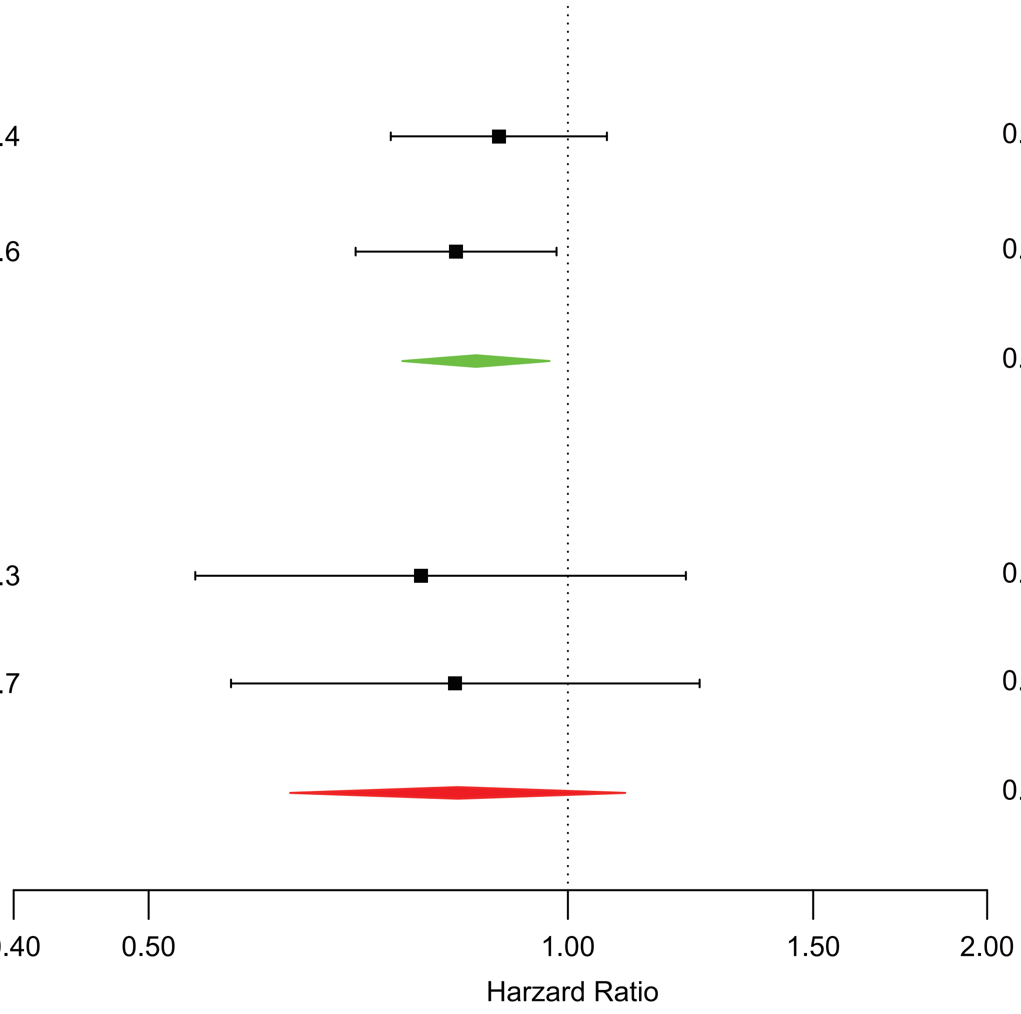

Supplement: Supplementary file 1 — Supplementary Material 1 [file 13098_2023_1251_MOESM1_ESM.pdf]
